# Alzheimer's Disease as a Result of Stimulus Reduction in a GABA-A-Deficient Brain: A Neurocomputational Model

**DOI:** 10.1155/2020/8895369

**Published:** 2020-10-14

**Authors:** Mariana Antonia Aguiar-Furucho, Francisco Javier Ropero Peláez

**Affiliations:** ^1^Engineering, Neuroscience and Bio-Inspired Systems Study Group (GENeSis), Department of Electrotechnics (DAELT), Universidade Tecnológica Federal do Paraná (UTFPR), Paraná 80230-901, Brazil; ^2^Center for Neuroscience and Behavior, Institute of Psychology, University of São Paulo, São Paulo, Brazil; ^3^Center of Mathematics, Computation and Cognition (CMCC), Universidade Federal do ABC, Brazil

## Abstract

Several research studies point to the fact that sensory and cognitive reductions like cataracts, deafness, macular degeneration, or even lack of activity after job retirement, precede the onset of Alzheimer's disease. To simulate Alzheimer's disease earlier stages, which manifest in sensory cortices, we used a computational model of the koniocortex that is the first cortical stage processing sensory information. The architecture and physiology of the modeled koniocortex resemble those of its cerebral counterpart being capable of continuous learning. This model allows one to analyze the initial phases of Alzheimer's disease by “aging” the artificial koniocortex through synaptic pruning, by the modification of acetylcholine and GABA-A signaling, and by reducing sensory stimuli, among other processes. The computational model shows that during aging, a GABA-A deficit followed by a reduction in sensory stimuli leads to a dysregulation of neural excitability, which in the biological brain is associated with hypermetabolism, one of the earliest symptoms of Alzheimer's disease.

## 1. Introduction

Since Alzheimer's disease (AD) is a complex, multifaceted illness (see Subsection 1.1), it is difficult to evaluate the relationship between the many factors involved (genetic, cognitive, social, sensory, neural, and molecular). This relationship should be sought at the level of the neural circuits that process information, either from the senses or from other areas of the brain. Neurons in these circuits are so tightly packed [1] that inserting electrodes in predetermined neurons to assess their operation is very costly. Even with the advent of optogenetics, which allows neurons to be activated by light [2], the task of studying circuit-level interactions of neurons, by changing their parameters and connectivity, is still a significant challenge that is easily tackled with neurocomputing models [3]. Neurocomputational modeling has strong theoretical support from the area of artificial neural networks (ANNs), in which different arrangements of neuron-like units contribute to the development of artificial intelligence (AI) systems. Although neural networks (NNs) models are far from being biological, they can be used to understand biological NNs. Concepts like neural competition, synaptic weight adjustment, activation-function shifting, vector separation, and pattern normalization contribute to understanding not only artificial but also biological NNs.

One of the most exciting applications of NNs models is the creation of brain disease models. Such a model can be developed by “lesioning” an ANN that in “healthy” conditions performs functions like learning, pattern completion, abstraction, generalization, and categorization.

According to the seminal book *Neural Modeling of Brain and Cognitive Disorders*, “recently, a new direction has emerged, that of using “lesioned” neural models to study several brain and cognitive disorders from a computational point of view” ([4], p.3).

In 1996, when this book was published, there were well-developed models of memory, generalization, and categorization implemented in artificial NNs. However, there was still a lack of knowledge about how these operations take place in real neural circuits. For this reason, earlier computer models of neurological disorders made use of conventional artificial NNs models rather than biologically plausible neural circuits. Even with the lack of biological realism, these first computer models attempted to model amnesia [5], dyslexia [6, 7], stroke [8], phantom limbs [9], Parkinson's and Huntington's diseases [10], schizophrenia [11], and even AD [12, 13]. Over time, with an increasing understanding of biological neural circuits, realistic neural models of AD appeared [14]. While some of these models tried to emulate the hippocampal function [15, 16], others like Stefanovski et al. model [17] performed whole-brain simulations for inferring candidate mechanisms of AD.

Realistic neurocomputational models endowed with operations at molecular levels can be very costly in computational terms. On the other hand, the so-called phenomenological models (see Section 1.2 in [18]) are simplified models that try to capture the minimum characteristics capable of simulating the basic operations of the modeled phenomenon. In phenomenological models, the focus is the function, not the details of the substrate in which the activity takes place. For example, in the case of biological neurons, they are modeled by mimicking their input-output operations without regard to molecular complexities. We adopted this latter type of model for our simulations.

Initially, instead of simulating brain impairment, our priority was to develop operational models of brain structures like the thalamus [19, 20], the amygdala [21], and the koniocortex [22, 23]. When their initial operation was satisfactory, the way of further evaluating them was by modeling brain disorders. For example, in a previous paper [24], incomplete sensory patterns when entering the artificial model of the thalamus yielded a reconstructed copy reminiscent of hallucinations in actual schizophrenic patients. This result led to propose a correlation between the genetic lack of thalamic afferents from prefrontal and temporal areas [25] and the so-called positive symptoms of schizophrenia. A similar strategy allowed the assessment of the circuit linking the thalamus and the amygdala, which led to an effective therapy for treating specific phobias [21]. We also modeled the koniocortex, the first cortical layer receiving inputs from the sensory thalamus [23, 26]. Once modeled, learning emerges competitively with spiny stellate (SS) neurons behaving in a “winner-take-all” (WTA) manner (In a pool of neurons, “winner-take-all” means that the most activated neuron starts firing while the others keep silent.). The artificial koniocortex performs stimuli (input patterns) classifications the same way competitive NNs do (i.e., by firing a single neuron for each category of stimuli presented to the network).

As will be shown in Subsection 1.2, there is a strong positive correlation between the onset of AD and stimulus reduction in the nervous system. This stimulus reduction can be of two types:

- A sensory reduction like in macular degeneration, deafness, or cataracts
- Cognitive task reduction, like in retirement, loss of employment, or loss of or separation from relatives

Since the koniocortex model performs memorizing tasks, simultaneously dealing with input patterns, it seems the right candidate for testing the hypothesis correlating to the AD onset and the reduction of sensory stimuli. Furthermore, cortical sensory areas like the koniocortex are also the first ones to be affected by AD [27]. This fact was not so evident at the beginning of AD research, due to the difficulty in identifying amyloid plaques in these areas because they appear in their mildest diffuse or amorphous forms. Beach and McGeer were able to highlight these diffuse plaques by using the Bielschowsky stain technique [28]. Recently, advanced technologies like Spatial Proteomics Analysis have not only shown AD-related protein alterations at sensory cortices but also demonstrated that the evolution of AD begins in sensory and motor cortices where the disease appears in its mildest forms [29]. Other studies give further support to these findings and show that SS neurons in the koniocortex undergo significant density decrements and dendritic loss during aging [30] and AD [31].

As mentioned previously, biologically plausible computational models can be tested by “lesioning” them in ways similar to the lesions experienced in their biological counterparts. For assessing whether the koniocortex model mimics Alzheimer's symptoms, we simulate its “aging” by altering its parameters. Although this “aging” operation seems somewhat unspecific, the main factors of nervous system aging are not unknown to scientists (see Subsection 1.3). They might be reproduced in a modeled neural circuit, as will be explained.

One property of neurons in the koniocortex model that is essential for learning processes is called intrinsic plasticity (IP) [32, 33]. IP dynamically adjusts neurons' firing threshold performing a dual-type operation (see the appendix):

- In the case of very active neurons, IP makes the neuron's firing threshold more positive for lowering its firing rate in the future. Neurons do this by eliminating intrinsic channels (like L-type Ca^2+^ channels, Na^+^ channels, and delayed rectifier K^+^ channels) from the neuron's membrane ([33], Section 4)
- Conversely, in the case of low neuronal activity, IP makes the firing threshold less positive, which increases the firing rate in the future. Neurons do this by placing intrinsic channels in the cell membrane. Porter et al. [34] associates the excessive expression of calcium channels in the neuron's membrane to neurotoxicity and AD

**Figure 1:**
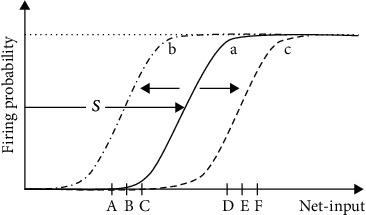
Intrinsic plasticity. (a) The continuous line at the center represents the activation function at a hypothetical initial state. (b) When the net-input values are diminished as in A, B, and C, the sigmoidal activation function shifts leftwards. (c) When the net-input values grow like in the case of D, E, and F, the sigmoidal activation function shifts rightwards.

In NNs models, the position of the firing threshold roughly coincides with the rightwards shift of the neuron's activation function (see Figure 1) that in many cases is “s”-shaped (or sigmoidal) like the one used by Desai ([33], Fig. 3.a) to explain IP. In neurocomputational models, the terms “shift” and “firing threshold” are interchangeable. The value of the shift is between zero and one: zero corresponding to the minimum firing threshold and 1 to the maximum firing threshold.

IP is a homeostatic process in which the shift/threshold tends to follow the average neural activity (ANA). Depending on the velocity in which this ANA moves, the average shift will quickly reach the ANA, or it will dynamically oscillate around it until eventually catching it up.

This latter process might be related to the putative link (see Section 1.2) between the onset of AD and stimuli reduction, as may occur during job retirement or separation from relatives. When neurons suddenly diminish their activity due to stimuli reduction, IP initially operates by making the neuron's firing threshold less positive so that the neuron becomes more easily fired. Ideally, this process makes the neuron more active until it gradually stabilizes. However, in nonideal conditions, an oscillatory process around the ANA occurs. In this process, hyperactivation and hypoactivation alternate until the ANA and the shift match. Along this process, the neuron's IP adjusts the shifts by either creating or eliminating intrinsic channels. The continuous production/elimination of intrinsic channels makes use of significant metabolic resources leading to hypermetabolism (see Subsection 1.4.4). Hypermetabolism [35] appears in Magnetic Resonance Imaging (MRI) and Positron Emission Tomography (PET) scans of AD patients and is simultaneous with the stage of Mild Cognitive Impairment (MCI), in which patients experience difficulties in recalling recent memories. It also precedes the stage of beta-amyloid plaque proliferation (a review of all the stages of AD is presented in Subsection 1.4).

Since the koniocortex network is able to continuously learn new patterns during its regular operation, one can evaluate its learning performance over time. We initially use an integral (nondamaged) network and subsequently apply several types of damage associated with aging (see Subsection 1.3 and Table 1). Finally, we reduce the intensity (module) of the input patterns. We will see that when this reduction takes place in a network with impaired GABA-A inhibition, a persistent oscillatory dynamic takes place. Although oscillations are an integral part of learning, persistent oscillations can disrupt learning. Continuous oscillations also lead to hypermetabolism. Since phenomenological models do not usually deal with molecular properties, our koniocortex model cannot directly assess hypermetabolism. Instead of this, to know whether the koniocortex model is reaching the stage of hypermetabolism, we continuously evaluate both the average output and the average shift of neurons. When these two markers engage in a persistent oscillatory dynamic, this fact determines the onset of the hypermetabolic stage.

Next, we provide more detailed explanations of some of the concepts presented in Introduction.

### 1.1. General Description

Characterized as a chronic, degenerative, and fatal disease, AD accounts for 60%-70% of dementia diagnoses worldwide [36] and is estimated to affect 106.2 million persons or 1 in 85 persons by the year 2050 [37]. The disease is partly hereditary, due to pathogenic genetic mutations, as well as external factors like dietary habits, and usually affects people over 65 years of age ([38], p.3).

Dietary patterns are a risk criterion as well as a factor of protection against the incidence of AD. Among the nutritional habits considered beneficial for reducing the risk of AD are those that stimulate the consumption of antioxidants, vitamins, polyphenols, fruits, vegetables, polyunsaturated fatty acids, fish, and tea, such as the Japanese and Mediterranean diets [39]. On the other hand, a large intake of red meat, butter, and high-fat dairy increases the risk of developing AD [40].

One of the first symptoms reported by patients and caregivers is difficulty in remembering facts, events, and names of people close to them. However, an AD diagnosis requires the presence of other concurrent problems like mental confusion, impaired executive functions, apathy, and communication difficulties [41]. According to Masters et al. ([38], p.9), the average clinical duration of AD is between eight and ten years, preceded by preclinical and prodromal phases, and a maximum span of 20 years.

Histopathologically, a key feature is a proliferation of senile plaques, aggregations of the insoluble form of the *β*-amyloid peptide (A*β*), not only in the entorhinal cortex, hippocampus, and associative cortices [42] but also, in a milder way, in sensory and motor cortices where AD starts according to Spatial Proteomics Analysis techniques [29]. Neurofibrillary tangles formed by the tau protein are also characteristic of the disease. They appear in cortical neurons, mainly in the entorhinal cortex, hippocampus, frontal cortex, and temporal and parietal lobes [42].

### 1.2. Principal Causes of Stimulus Reduction due to Age

Decreased sensory stimulation usually takes place due to the loss of sensory receptors or due to age-related health problems, such as macular degeneration, deafness, or cataracts. Retirement, a sedentary lifestyle, or the loss of relatives and friends could be external factors that contribute to decreasing the intellectual and cognitive stimuli experienced by older persons. Let us study these phenomena by classifying them into the following two types.

#### 1.2.1. Sensory Reduction

Macular degeneration, cataracts, or deafness are different ways in which sensory reduction appears in elders. Age-related macular degeneration (AMD) is a neurodegenerative disease that affects the macula (the central region of the retina), causing progressive loss of vision. AMD affects 15% of people between 65 and 74, 25% of people between 75 and 84, and 30% of people older than 85; it also shares many characteristics with AD, including oxidative stress and inflammation [43]. According to Kaarniranta et al. [43], studies on AMD are “interesting opportunities to understand the early signs of AMD that might be associated with AD pathology as well.” According to Javaid et al. [44], eye examinations allow an earlier diagnosis of AD because A*β* plaque deposition and hyperphosphorylated tau protein first appear in the retina. These authors point out that AD patients display an increased prevalence of cataracts affecting visual acuity.

Regarding deafness (which affects 30% of adults over 60), Lin et al. [45] show that early treatment for deafness postpones the onset of AD symptoms.

#### 1.2.2. Cognitive Task Reduction: Retirement, Loss of Employment, etc.

Using data from the Survey of Health, Ageing and Retirement (SHARE) in Europe, Adam et al. [46] examined whether the cognitive decline in aging could be affected by occupation or more specifically by inactivity after retirement and the relationship of these variables to participants' physical and mental health. The research revealed that retirees or individuals who never had a professional activity had lower performance on cognitive and occupational tests compared with professionally active participants ([46], p.385). Furthermore, retired people who engaged in cognitively stimulating activities or had social or religious involvement performed better than those who did not [46].

Lupton et al. [47] showed that late retirement acts as a protective factor against AD by postponing its age of onset, while the education level or type of occupation had no effect. In line with those results, Grotz et al. ([48], p.9) showed a strong positive correlation between the appearance of the first symptoms of AD and early retirement, indicating that postponing retirement by one year delays AD by 0.3 years.

Bonsang et al. [49] and Finkel et al. [50] confirmed that promoting the participation of older workers in the labor force delays cognitive decline and thus the occurrence of associated impairments.

### 1.3. Typical Nervous System Alterations due to Age

During normal aging, some neurophysiological changes impair long-term mnemonic systems and working memory: (a) the synaptic pruning of cortical neurons [51], (b) a reduction in the synthesis and release of acetylcholine (ACh) [52], and (c) the attenuation of inhibitory signaling of GABAergic interneurons in the hippocampus and prefrontal cortex (PFC) [53]. Next, we explain these processes in more detail.

#### 1.3.1. Synaptic Pruning

Although synaptic pruning (also known as synaptic connection harvesting) is associated with AD, it also occurs at every stage of a healthy brain's development and maturation. Synaptic pruning obeys Lamarck's law: “use it or lose it” by keeping only the reinforced connections during learning. According to Gopnik et al. [54], there is a decay from 15,000 synaptic connections to approximately 7,500 synaptic connections per neuron in older individuals. In an expressive graph ([55], Fig. 3), Huttenlocher depicts the evolution of synaptic density (in synapses per cubic milimeter) in the middle frontal gyrus as a function of age. It shows that this density increases from birth to around five years of age. From that point, synaptic density decreases until it stabilizes at around the age of 40 and starts falling again (about 75 years), linearly, until death.

Using mutant APP (beta-amyloid precursor protein) mice, Bezprozvanny and Mattson [56] showed a correlation between AD and synaptic pruning: the appearance of toxic forms of *β*-amyloid peptides (present in AD) is correlated with the loss of synaptic spines.

An article published by Nikolaev et al. [57] discusses the relationship between A*β* protein, “death receptor 6” (DR6 or TNFRSF21), synaptic pruning, and neuronal cell death. The DR6 receptor triggers cell death in response to low cell growth factor levels at specific periods of brain tissue development or when this tissue is damaged. The authors also present a loss/gain function model in which a fragment of the A*β* protein would bind to the DR6 receptor. This binding triggers neuronal degeneration and the self-destruction observed in AD. According to Nikolaev et al. [57], this mechanism occurs due to genetic causes or to the decrease in cell growth factors found in aging brain tissue.

A study by DeKosky and Scheff [58] supports the findings of Nikolaev et al. [57] by showing that the postmortem brain tissue from the right frontal lobe of patients with a mild form of AD exhibits decreased synaptic counts with an increase in the remaining contact area, compared with a control group. This fact suggests, according to the authors, that there is a “law of compensation,” aimed at maintaining the total contact area of the synapses per unit volume at a stable level. However, this ability is lost throughout the progression of the disease. In its final stages, both the number of synapses and the total area of synaptic contact suffer a significant loss that negatively affects patients' cognitive capacity.

Horn et al. ([12], p.737) cite DeKosky and Scheff [58] in the development of their computational model of memory decay due to the gradual and progressive deterioration of synaptic connections during AD evolution, presenting a “framework for examining the interplay of synaptic deletion and compensation” [12].

#### 1.3.2. Acetylcholine Deficit

Acetylcholine (ACh), one of the most abundant neurotransmitters present in the human brain, is directly involved with neural excitability, hippocampal-dependent learning [59], and memory processes [60]. Martinello et al. [59] demonstrate the importance of ACh for synaptic communication and, consequently, for memory formation.

The excessive neuronal loss characteristic of AD occurs mainly in cholinergic neurons of the basal forebrain (BFCN), which are also susceptible to axonal alterations, accumulation of phosphorylated tau protein, and formation of neurofibrillary tangles [61]. This set of factors led to the cholinergic hypothesis of AD. Francis et al. [62] proposed that an individual with AD presents degeneration of cholinergic neurons, a decrease in the activity of choline acetyltransferase (ChAT) and acetylcholinesterase (AChE), and reduction of ACh levels and cholinergic transmission mechanisms. According to them, these factors cause the cognitive impairment characteristic of the disease [63].

One of the treatments used in the earliest stages of AD includes drugs that act on cholinergic centers [62, 64], particularly on cholinesterase inhibitors. Although they contribute to improving the cognitive and behavioral aspects of AD, these medications do not prevent disease progression [64].

Although at first an ACh deficit seems to be one of the leading causes that disrupt the normal functioning of neuronal activity in AD, studies indicate that GABA-A may also play a critical role in the development of the disease [65].

#### 1.3.3. GABA-A Deficit

GABAergic inhibitory interneurons (GABA: gamma-aminobutyric acid) play a crucial role in the regulation of neural dynamics. According to McQuail et al. [53], imbalances in this system might result in psychiatric damage and neurodegenerative diseases such as AD.

Although researchers initially asserted that GABAergic neurons are relatively preserved during aging, and in neurodegenerative pathologies, recent research shows that the GABA-A type undergoes significant changes due to age and may play a primary role in AD [65]. According to Limon et al. [66], there is a profound loss of GABA-A receptors in AD.

GABA-A receptors are ionotropic, contain intrinsic channels permeable to chlorine (Cl^−^), and participate in most of the inhibitory connections of the brain through shunting inhibition [53]. Shunting inhibition is characterized by “an increase in conductance, leading to a reversal potential near the chlorine resting potential” ([67], p. 136). Shunting inhibition involves the entry of negatively charged Cl^−^ into the neurons, which hampers action potential firing, thereby resulting in a mathematically divisive effect on cell depolarization [68] (this effect will be explained when presenting shunting basket neurons in Section 2 and also in Equation (A.3)).

Nowadays, there is an increasing interest in looking for nutrients that contribute to the synthesis of GABA-A receptors. According to Currais et al. [69], fisetin (present in fruits and vegetables like strawberries, tomatoes, oranges, and cucumber) enhances mnemonic systems in healthy individuals. It mitigates the cognitive decline characteristic of neurodegenerative diseases such as AD. In line with this, Raygude et al. [70] showed that administering fisetin increases GABA-A levels in the brain. Another promising set of substances that contribute to the expression of GABA-A receptors in GABAergic synapses are terpenoids [71]. Terpenoids are found in vegetables and spices like salvia, peppermint, ginger, Curcuma longa, cinnamon, cloves, and mustard. Used in traditional medicine [72, 73] for improving cognitive functions, several authors cite terpenoids as promising therapeutic substances against AD [74, 75].

### 1.4. Stages of Alzheimer's Disease

#### 1.4.1. Preclinical

The preclinical phase occurs approximately 12 years before the onset of symptoms. In this stage, the patient does not usually exhibit signs of dementia. Masters et al. [38] consider this phase a window for disease prevention because it is rather lengthy and exempt from severe cognitive impairments.

According to Masters et al. ([38], p.9), at this stage, there is an increase in levels of the main AD biomarkers, such as *β*-amyloid binder protein (A*β* deposition) and isoform 42 of the *β*-amyloid protein (CSF A*β*42). This latter biomarker can be identified in the cerebrospinal fluid (CSF) 20 years before the onset of the first symptoms.

At this stage, approximately 15 years before the onset of disease symptoms, there is also an increase in the level of tau protein in the cerebrospinal fluid (CSF tau) exceeding normal thresholds.

Reduced hippocampal volume, Clinical Dementia Rating-Sum of Boxes (CDR-SB) scores, and glucose metabolism levels (hypermetabolism at the preclinical stage and hypometabolism in the transition phase between the prodromal and clinical stages) are also considered important biomarkers of AD.

#### 1.4.2. Prodromal

The prodromal phase begins with the sudden manifestation of cognitive symptoms related to dementia and memory loss at a level below that associated with AD ([38], p.9). High levels of disease biomarkers begin during this phase and extend towards the mild-to-moderate stage.

#### 1.4.3. Mild Cognitive Impairment Stage

The Mild Cognitive Impairment (MCI) stage of AD occurs after the prodromal phase. The MCI stage is considered a preclinical phase because the changes observed in memory and cognitive functions differ from those considered normal during the aging process [76].

There are two types of MCI: (a) Simple Domain Amnestic MCI, which affects only memory, and (b) Multiple Domain Amnestic MCI, which affects memory and one or more cognitive functions like language, attention, perception, or executive functions. Busse et al. [77] reported that Multiple Domain Amnestic MCI can be considered a preclinical stage of AD.

Recently, the National Institute on Aging and the Alzheimer's Association have revised the criteria for the diagnosis of MCI as a preclinical indication of AD [78] and have recommended additional procedures for brain assessment, including testing biomarkers for A*β* and brain imaging.

#### 1.4.4. From Hypermetabolism to Beta-Amyloid Plaque Creation

Hypermetabolism (increased glucose metabolism observed with fMRI and PET scans) occurs in subjects with MCI before the development of beta-amyloid plaques [35]. This fact is supported by Dickerson et al. findings [79], who reported increased hippocampal activation in MCI subjects. Busche et al. [80] studied individual cortical neurons in a mouse model of AD and reported increased neuronal activity in the direct vicinity of A*β* plaques. These authors suggest that this increased activity may also contribute to the calcium overload recently observed in neurites surrounding A*β* plaques. Regarding hypermetabolism and beta-amyloid plaque creation, Kim et al. [81] noticed that metabolism (measured by the uptake of FDG [18F] fluoro-2-deoxyglucose in the basal forebrain region) was higher in patients in the early stages of the disease and in MCI patients than those already diagnosed with AD and healthy subjects. According to those authors, this metabolic increase may be responsible for *β*-amyloid plaque formation leading to dementia ([81], p.935).

These metabolic changes, as well as perturbed calcium homeostasis, support the idea that Alzheimer's disease is related to mitochondrial dysfunction [82].

Finally, one of the latest manifestations of AD is hypoactivity, which is due, according to Bass and colleagues [83], to a combination of homeostatic alterations and A*β* plaque proliferation. As mentioned in Introduction, our computational model is not able to simulate the A*β* plaques proliferation, being only able to predict events until reaching the initial hypermetabolic stage that is associated with persistent neural oscillations.

## 2. Materials and Methods

As mentioned in Introduction, this research study is based on a computational model of the cerebral koniocortex that we developed in previous work [23, 26].

The term koniocortex, also known as granular cortex, means a cortex with a grainy texture (Konia “dust” in Greek) due to the high density of spiny stellate (SS) neurons. It refers to the different cortical regions with a distinctive inner granular layer (layer IV). The koniocortex includes Brodmann areas 1-3 (somatic sensory cortex), 17 (visual cortex), and 41 (auditory cortex). All these areas are like topographic maps that undergo plastic changes in their boundaries and receptive fields according to sensory experience. These changes are mainly due to NMDA receptors in koniocortex spiny stellate (SS) cells [84].

**Figure 2:**
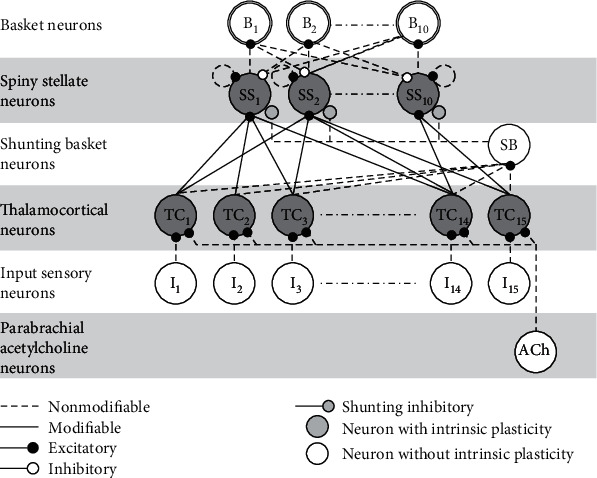
Cytoarchitecture of the koniocortex network in which acetylcholinergic neurons from the parabrachial nucleus project to thalamocortical neurons. The neurons that specifically belong to the koniocortex are the spiny stellate (SS) neurons, the inhibitory basket neurons (B), and the shunting basket (SB) neurons.

The cytoarchitecture of the koniocortex is depicted in Figure 2. SS neurons receive excitatory and inhibitory afferents, each of which has two types.

**Figure 3:**
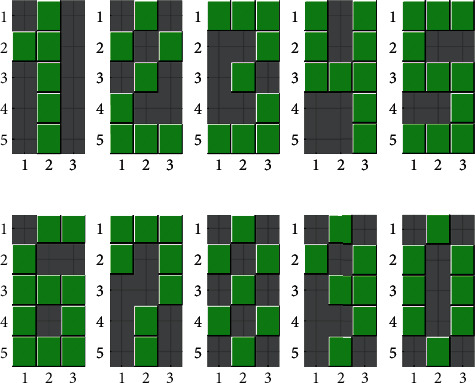
Alphanumeric input patterns presented as inputs to the koniocortex model.

(1) Excitatory afferents:
  - Autapses [85]
  - Afferents from thalamocortical neurons (TC) in the thalamus that process sensory information
(2) Inhibitory afferents:
  - Basket neurons (B) that are stimulated by nearby excitatory SS neurons and exhibit the steepest activation-function slope among all neurons in the koniocortex ([86], Figure 3)
  - Shunting basket neurons (SB), which, according to Angulo et al. [87], accomplish linear summation of their thalamocortical afferents. In mathematical terms, this operation is called l1-norm (see Equation (A.2) in the appendix). These neurons use this result to produce a type of shunting/divisive inhibition ([88], p.1225) over SS neurons. This divisive inhibition is due to the GABA-A receptor used in shunting basket neurons' axon terminals. The concatenation of these two operations (the l1-norm and the division) means that shunting basket neurons perform a sort of normalization in their targets, the SS neurons (see Equation (A.3) in the appendix)

As previously mentioned, we developed a phenomenological model of the koniocortex in which we took into account the main functionalities of each of the neurons. Each neuron communicates with the following one by transmitting its output, a value between 0 and 1 that in spiking models corresponds to the neuron's firing rate. This value, when multiplied by a synaptic weight, is the synaptic contribution to the next neuron (see the appendix explaining the mathematical background).

The koniocortex model can learn how to classify input patterns like a conventional competitive NN. Although the artificial koniocortex deals with input patterns of any size, we used patterns represented in a 5 × 3 grid (Figure 3), a total of 11 alphanumerical patterns. Ten patterns were numbers (0 to 9), and one was a letter (X). These patterns were forcedly placed at the output of the 5 × 3 = 15 sensory input neurons (I) of the koniocortex model (see cytoarchitecture depicted in Figure 2).

The learning processes take place in our simulations along 2,000 epochs, that is to say, 2,000 repetitions of the complete set of patterns that represent the life span of our model. Within these 2,000 epochs, we will model several natural processes taking place in the human brain from birth to death. Before 50% of repetitions, ten SS neurons of the koniocortex compete to recognize each one of the numerical patterns, so that, in the end, a single neuron fires for each one of the presented numbers. This type of specificity occurs because the synaptic weights of each neuron evolve to reflect the distinctive characteristics of each numerical pattern. In the end, when the synaptic weights of each neuron match the unique features of each number, the firing of a specific neuron takes place.

Although the synaptic weights of each SS neuron evolve to match the differential characteristics of each input pattern (not the complete input pattern), we still would like to recover the entire numerical pattern that fires each neuron for assessing the correctness of the pattern classification. For this purpose, it was necessary to create a recurrent ancillary network consisting of a set of virtual feedback connections ([26], Figure 6(b)) from spiny neurons to sensory neurons. At the end of the training, the collection of virtual weights exiting each SS neuron recreates the whole pattern that produces the firing of each spiny neuron. We will use this strategy for recalling the numerical pattern associated with each spiny neuron throughout training and test whether the modeled AD affects stored memories. In Results, we will see that when simulating AD, the numerical patterns (recalled through these virtual connections) degenerate, and neurons lose their specificity, firing in front of more than one number.

When inputting each numerical pattern (by making numbers appear at the output of the sensory neuron's layer), this input information should spread until reaching the SS neurons. From the SS neuron's layer, the information propagates towards both the basket neurons' layer and the input neurons' layer (to the latter through the recurrent connections of the ancillary network). For allowing sensory information propagates across all layers, thereby producing a competitive interaction between spiny neurons, each numerical pattern should remain at the input during eight iterations. In each iteration, the program calculates the neuron's outputs, weights, and shifts, according to Equations (A.4) to (A.7) in the appendix. It is important to emphasize that the koniocortex network and the recurrent ancillary network update simultaneously (all their weights and shifts update at the same iteration). It is as if both networks constituted a single associative network of the type shown in ([26], Figure 9).

In the present simulations, patterns were input to the network sequentially, although they could also appear randomly. We found that when patterns are sequential, autapses are not necessary for a correct learning process. However, for the emergence of competitive learning in the case of random patterns, autapses are required (see the last paragraph of Section 3.3 in [26]).

In this simulation, the learning factor *ξ* (Equation (A.4)) was set to 0.0019, and the shifting velocity *υ* (Equation (A.7)) to 0.0199. We obtained these optimal values by using a genetic algorithm for optimizing WTA processes in the SS neuron's layer. Nonmodifiable weights were set to *W*_SS−SB_ = 0.98, *W*_I−TC_ = 1.0, W_SB−SS_ = 0.5, and *W*_SS−SS=_0.85 in the autapses. Modifiable weights from TC to SS neurons and from SS to I neurons of the ancillary network start with negligible random values. The steepness factor *k* in the sigmoid of all neurons except for basket neurons was set to *k* = 40. The activation function of basket neurons (that have the steepest activation) is linear. All the sigmoidal function shifts were set initially to 0.061.

In Results, we will analyze the computational processes taking place in the koniocortex model that, when disrupted, might lead the model to behave like the brain of patients in the initial stages of AD (see Table 2). We will do this by using the point of view of an ANN designer. Although most ANNs are biologically implausible, some theoretical aspects of ANNs are valid for any NN, either artificial or biological. For example, we will study two crucial computational processes mentioned in seminal treatises [89, 90]. The two computational processes necessary for achieving successful learning in competitive NNs are

- input pattern separation
- input pattern normalization

**Figure 4:**
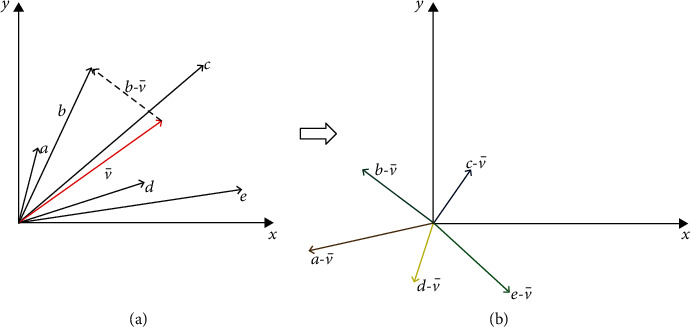
(a) One way of emphasizing the distinctive features of a set of vectors *a*, *b*, *c*, *d* consists in subtracting their average vector (as in the case of vector *b*). (b) In this way, vectors become more separated (in terms of the angle between them).

Regarding the first, when the angular separation between input patterns is small, a winning neuron can win again for many other input patterns, thereby precluding other competitive neurons from winning. One way of separating input patterns and preserving their distinctive features is to subtract their mean (also called moving average), as shown in Figure 4.

In the case of the koniocortex model, this process occurs at the level of thalamocortical neurons. This is related to IP, explained in the appendix. According to Peláez et al. [26], “Intrinsic plasticity is also highly important at the thalamocortical neuron level (second layer). In this layer, intrinsic plasticity contributes to subtracting the average neuron's activity level from current activity. The previous assertion means that the average pattern is subtracted from each incoming pattern, thus contributing to highlighting the differences between input patterns.”

According to Martinello et al. [59], ACh from the parabrachial nucleus induces IP in thalamocortical neurons, thereby boosting the pattern separation process described above. An age-related cholinergic impairment might hinder this separation process so that, in the end, input pattern separation no longer occurs.

The second computational process (see “computational process” column in Table 2) for achieving successful learning in the koniocortex is normalization. As mentioned in Subsection 1.3.3, shunting basket neurons (SB in Figure 2) produce shunting/divisive inhibition of SS neurons through their GABA-A synapses [88]. As explained in [87], prior to this process, SB neurons perform a linear summation of their thalamocortical afferents. These two operations, division, and summation participate in a normalization process that consists of dividing the weighted sum of thalamocortical outputs by their l1-norm (the sum of thalamocortical outputs), according to Equation (A.3) in the appendix. This normalization of neuronal input patterns is necessary for a fair competition between spiny neurons. Shunting basket neurons are, therefore, responsible for the process of normalization in the koniocortex model (see the “computational process” column in Table 2).

This normalization process, when damaged (see “age-dependent damage” and “computational failure due to age” in Table 2), can impair competitive learning in the koniocortex model.

Another computational process that is important for allowing learning processes in the koniocortex model is the competition between SS neurons due to lateral inhibition (see Figure 2). Basket neurons (whose activation function was modeled as a linear function without IP) are involved in this process by performing a conventional subtractive type of inhibition.

Regarding learning, it is not possible without functional NMDA channels in stellate neurons' spines. For keeping memories intact in NMDA synapses, even at the risk of precluding newer learning processes, NMDA synapses can be “frozen” with NMDA blockers such as memantine, as mentioned in the last column.

The other computational process impaired due to age is the input vector separation process occurring in thalamocortical neurons (recall comments to Figure 4). If, according to Martinello et al. [59], ACh boosts IP, the reduction of ACh would negatively affect IP by slowing down sigmoid shifting. When the sigmoid is unable to follow the ANA (recall introduction), this lack of synchronicity adversely affects pattern separation and competitive learning. However, many competitive networks do not use this preliminary process of pattern separation and still do their job. Consequently, we do not expect that slowing down sigmoid shifting (by reducing or zeroing factor *υ* in Equation (A.7)) will significantly impair learning.

The following is our eight-stage protocol:

1. Initially, the network's goal is to learn ten different numerical patterns (Figure 3). Learning takes place when each SS neuron fires only in front of one specific numerical character. Simultaneously, each SS neuron produces a copy of each current pattern, as explained in this section. If the copy is identical, learning is Ok. The more different the copy, the more unsuccessful is the learning process
2. At half the number of repetitions, we “age” the network by either simulating GABA-A impairment or a lack of ACh. GABA-A impairment is modeled by eliminating the normalization operation performed by shunting basket neurons. The lack of ACh that precludes the sigmoid shift is simulated, as previously explained, by setting parameter *υ* to zero in thalamocortical neurons
3. At 60% of the total amount of repetitions, we apply stimulus reduction by dividing each component of the numerical patterns by two (“pixel value”/2).
4. At 70% of the repetitions, we evaluate network performance using a short-term memory task. In this case, one of the numerical patterns (randomly selected) is replaced by the letter “X.” The network's performance during the task of learning this new pattern can be evaluated by the reader by conferring the numerical learned by each neuron (as explained in stage 1)
5. In some simulations (at 55% of the repetitions), we test the situation of applying an NMDA blocker (like memantine). We perform this test by preventing synaptic weight modifications
6. To assess hypermetabolism, we calculate the average output of all modeled neurons (not counting sensory neurons) over time. Hypermetabolism takes place when the average output is persistently oscillatory
7. We also assess the temporal evolution of the average neurons' firing threshold, that is to say, the average shift (not counting sensory neurons). Hypermetabolism is associated with average shift changes because these changes involve intrinsic channel creation and elimination
8. When the number of iterations is higher than 75%, synaptic pruning (see Section 1.3.1) starts running in the network. According to Huttenlocher [55], at the age of 74 (after a long period during which synaptic density stabilizes around 11.05 × 10^8^ synapses/mm^3^) synaptic density begins a steady decay at a rate of 18.63 × 10^6^ synapses/mm^3^ per year. Translating these values to our artificial model, at 74% of the iterations, we start a pruning process in which 1.7% of the synapses (those with the smallest synaptic weights) are pruned at each iteration

## 3. Results

The koniocortex model used here for testing the AD has fifteen neurons in its input layer, fifteen neurons in its thalamocortical neurons' layer, ten neurons in the spiny stellate neurons' layer, and ten neurons in the upper basket neurons' layer (Figure 2). The learning process of the koniocortex model allows the recognition of a set of 10 numerical patterns. A 5 × 3 grid displays these numbers. All these numeric patterns are presented sequentially to the network in each epoch. Once each number is input to the network, its activation is “propagated” until all layers are activated. One thousand epochs were enough for the NN to learn that when a specific SS neuron strongly fires in front of one particular numerical pattern, the remaining neurons should remain inactive. This WTA process occurs naturally as an emergent consequence of the individual computation of each neuron without the need to monitor the network externally. Lateral inhibition and IP are the main driving forces for the emergence of this WTA process.

**Figure 5:**
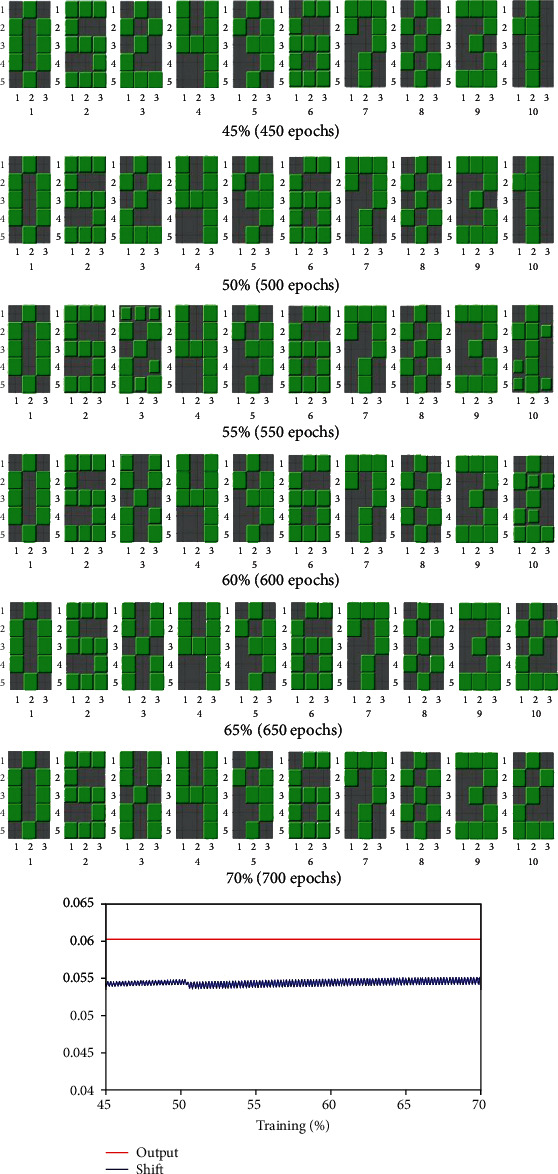
Example of koniocortex continuous learning under physiological (not defective) conditions. Each of the six rows represents the learning status at a selected epoch (iteration), being 1,000 the total number of epochs. In each of the six rows, ten synaptic weight matrices are corresponding to each one of the ten spiny neurons in the simulation, being each spiny neuron identified by a number below each matrix. The relative size of the fifteen green tiles in each matrix corresponds to the relative value of the weights of the recurrent virtual connections from spiny to input neurons. When, at epoch 500, one of the training patterns, pattern one, was substituted by a new pattern, pattern X, there is a process of weight reorganization for deciding which spiny neuron will fire in front of pattern X. At the end (see rows corresponding to epochs 650 and 700), spiny neuron three fires in front of pattern X and spiny neuron ten fires when pattern two is presented to the koniocortex model. In the bottom graph, we depict two curves: the red curve is the average output and the blue curve the average shift along with iterations. Both curves exhibit stable and regular behaviors.

To computationally test AD, we added one thousand additional epochs. These repetitions were intended to simulate the reduction of sensory stimuli and the GABA-A and ACh deficiencies that are customary in an aged brain. We will also evaluate how the network behaves in a continuous learning task, after substituting one of the patterns by a completely new one, an “X” pattern in the middle of typical training. We intend to do this experiment under defective conditions of the network so that we could evaluate how the capacity of continuous learning of the network is affected by the different types of impairment. For comparison purposes, we first present to the reader the same experiment under physiological conditions (without any kind of impairment). Each row of Figure 5 represents the results of training the koniocortex model along with a certain number of iterations (epochs). It shows that each one of the ten spiny neurons becomes specialized in recognizing a specific numerical pattern. This fact means that, when we present a numerical pattern to the network, one single spiny neuron “fires” (i.e., is active) while the other neurons remain muted. For example, only neuron “one” fires when pattern zero is presented at epoch 450 (see the first case in the first row). When spiny neuron “one” fires, it “evokes” number zero in the form of a green pixels' matrix, each pixel corresponding to the weight of a recurrent connection from itself (spiny neuron 1) to the input neurons. This process occurs at the recurrent ancillary network mentioned in Section 2. In the case the matrix was ambiguous or defective, it would mean that either a change of pattern or a memory problem is occurring in that neuron.

In this physiological example, when we reach 50% of the total number of epochs (500 epochs), we remove a numerical pattern, in this case, pattern 1, from the training set, and put the letter X in place. At epoch 550, we see that spiny neuron three and spiny neuron ten are “evoking” unclear, ambiguous patterns. This fact means that neurons are readjusting their weights for recognizing the new pattern X and forgetting the older pattern one. At epoch 600, the weights of spiny neuron 3 (that formerly “evoked” pattern two) evolve to represent pattern X. At epoch 600, the weights of spiny neuron 10 are still under transformation. This transformation is complete at epoch 650 when the recurrent weights from spiny neuron 10 evolve to represent pattern two. In this way, neuron ten that previously fired in front of pattern one now fires when pattern two is input to the network. At the same time, neuron three that fired when pattern two was input to the network, now fires in front of pattern X. At epochs 650, and epoch 700, the property of continuous learning of the koniocortex allowed the network to forget pattern 1 and learn pattern X. The bottom graph exhibits two curves, one curve in red and the other in blue representing the evolution of the average output and the average shift, respectively (without including sensory neurons). Both curves are stable and regular, although the blue curve exhibits small continuous oscillations.

As previously announced, this same short-term memory (STM) test will be performed in some of the simulations when training reaches 70% of epochs under nonphysiological (defective) conditions.

**Figure 6:**
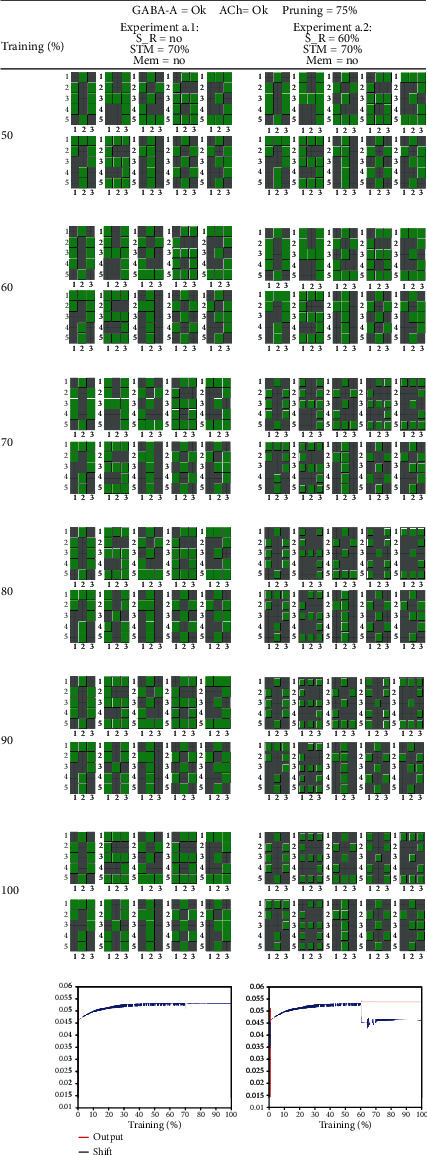
Normal aging. The two columns of this table show the koniocortex model behavior while performing a short-term memory (STM) task consisting of substituting one of the numerical patterns by pattern X. This substitution starts at 70% of epochs. In both experiments, the levels of GABA-A and ACh levels are normal, and pruning is also normal (starting at 75% of epochs). “Mem = no” means that in these experiments, we did not simulate the introduction of an NMDA blocker (memantine). At the bottom of each column, we show the evolution of the average output (in red) and average shift (in blue) of all neurons. Experiment a.1: the label “S_R = no” at the header of the first column means that there is no reduction of stimuli in this experiment. As seen in the block corresponding to 80% of training, the network successfully learns pattern X. Experiment a.2: the label “S_R = 60%” at the header of the second column indicates that there was a reduction of stimuli at 60% of repetitions. From this point, the average output and average shift experiment a sudden fall but stabilize rapidly. The presentation of a new pattern X at 70% of epochs does not alter the ongoing network dynamics in any significant way. Labels: S_R = sensory reduction; STM = short-term memory; Mem = memantine application; ACh = acetylcholine reduction in thalamocortical neurons.

**Figure 7:**
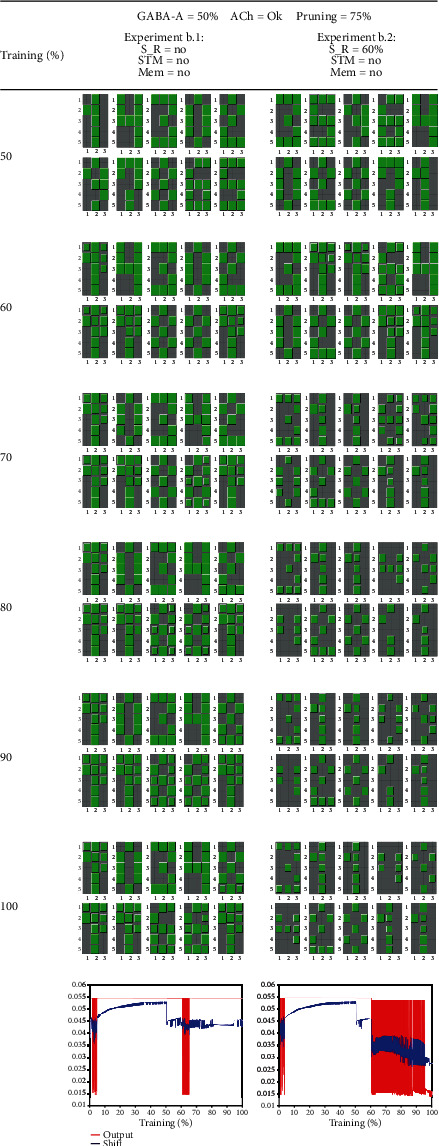
This table shows two cases without GABA-A signaling: the column on the left without sensory reduction and the column on the right with sensory reduction. At the bottom of each column, we show the evolution of the average output (in red) and average shift (in blue) of all neurons. Labels: S_R = sensory reduction; STM = short-term memory; Mem = memantine; ACh = acetylcholine reduction in thalamocortical neurons. In experiment b.1 (left column), there is no reduction of stimuli, and we see that, although an episodic period of acute output oscillations occurred, homeostatic mechanisms are capable of driving the network to equilibrium again. By examining the blocks, we see that pattern recall was permanently impaired. However, acute oscillations related to hypermetabolism and disease progression were extinguished. In experiment b.2, the withdrawal of GABA-A at 50% of epochs was the precondition for the production of intense oscillations when the sensory reduction took place at 60% of epochs. At the same time, at 60% of epochs, neurons lost their pattern specificity so that several neurons processed the same pattern (number 1). Sustained oscillations are associated with hypermetabolism and the progression of AD.

**Figure 8:**
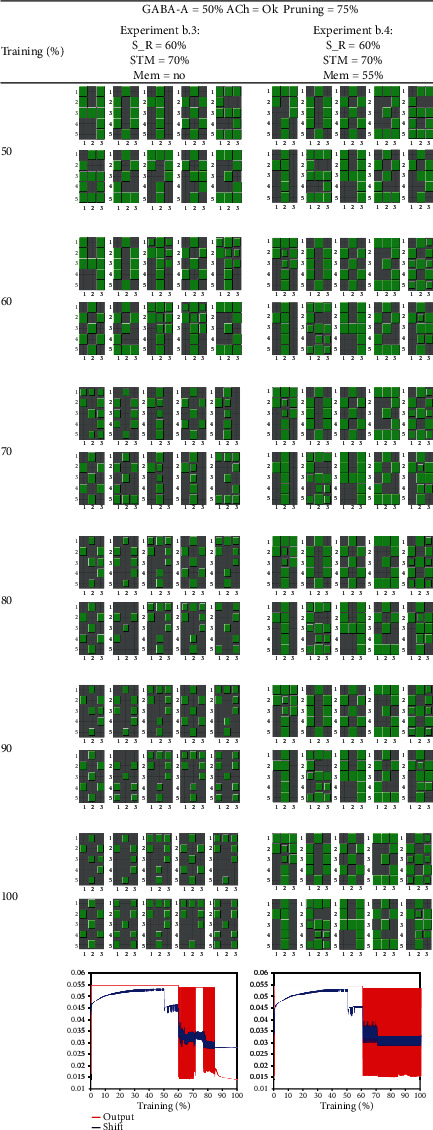
This table shows the koniocortex model behavior when the reduction of sensory stimuli takes place after a reduction in GABA-A. A short-term memory (STM) learning task is shown in the first and second columns, consisting of learning an “X” pattern in substitution of one of the numerical patterns that constitute the learning set. In the second column, we evaluate the usage of NMDA blockers like memantine. At the bottom of each column, we show the evolution of all neurons' average output (in red) and average shift (in blue). Experiment b.3: the pattern “X” presented at 70% of repetitions is successfully learned. Despite this apparent success, the univocal correspondence between patterns and neurons that were compromised at 60% of epochs did not return to normal. Notice that episodes of oscillation and stabilization are intermingled and that learning a new pattern stabilizes the network, although this stable situation is usually transitory. In experiment b.4, the administration of an NMDA blocker like memantine took place at 55% of epochs. Numerical pattern memories are kept intact, but, when introducing pattern “X” at 70% of repetitions, the network was incapable of learning it. Stimulus reduction at 60% in a GABA-A-depleted network contributed to the initiation of persistent oscillations. Labels: S_R = sensory reduction; STM = short-term memory; Mem = memantine; ACh = acetylcholine reduction in thalamocortical neurons.

**Figure 9:**
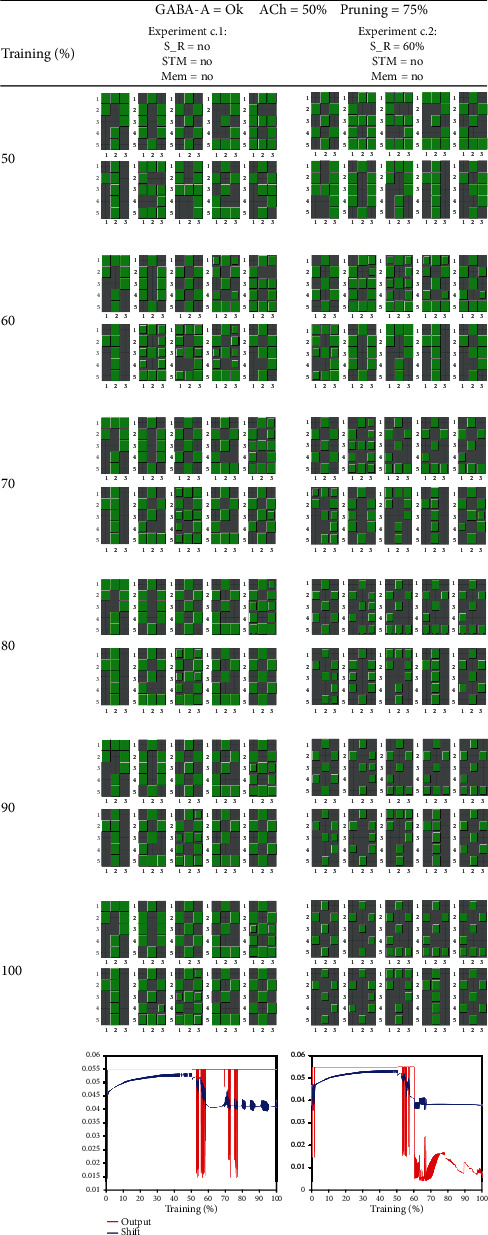
In these experiments, we reduce the effect of ACh at 50% of epochs. Experiment c.1, in which no other modification is involved, represents a control test for comparison with subsequent experiments. In this case, the bottom graph exhibits sparse oscillations of the average output. Experiment c.2, in which stimulus reduction takes place at 60% of epochs, produced smaller but sustained oscillations that are harmful to learning patterns. Labels: S_R = sensory reduction; STM = short-term memory; Mem = memantine; ACh = acetylcholine reduction in thalamocortical neurons.

**Figure 10:**
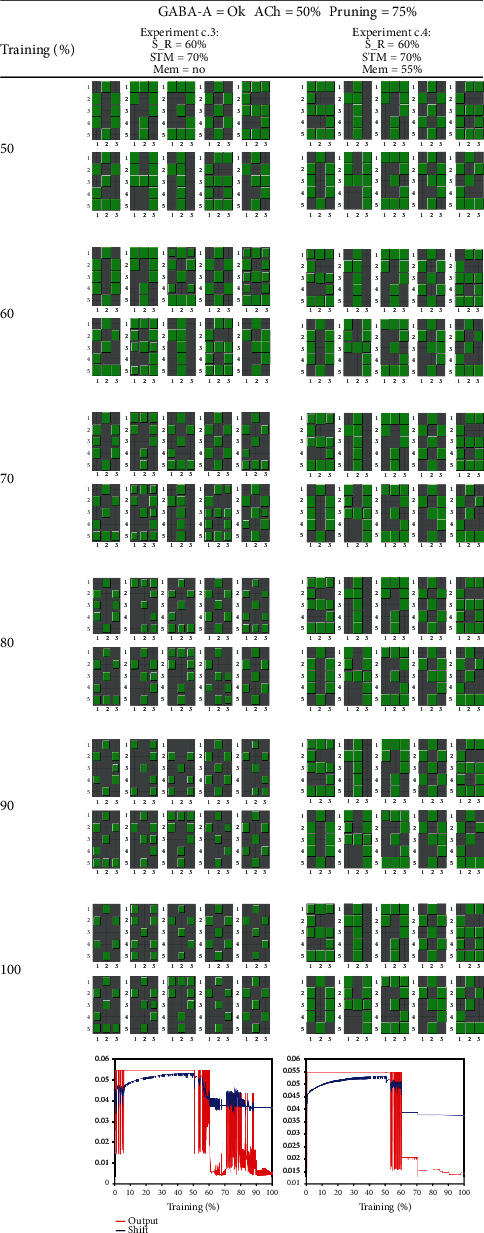
This table shows the koniocortex model response when GABA-A levels are normal, and we reduce ACh at 50% of repetitions. Sensory reduction also takes place at 60% of total repetitions, and a memory test is performed at 70%. Experiment c.3: an NMDA blocker (like memantine) is not used. Oscillations are present initially when ACh is reduced and, especially afterward, during the process of learning the new pattern X. Experiment c.4: an NMDA blocker (like memantine) is applied at 55% of epochs. In this case, plasticity is eliminated; the network remains at its former stability level (without oscillatory activity) although it is not able to learn the testing pattern X. Labels: S_R = sensory reduction; STM = short-term memory; Mem = memantine; ACh = acetylcholine reduction in thalamocortical neurons.

**Figure 11:**
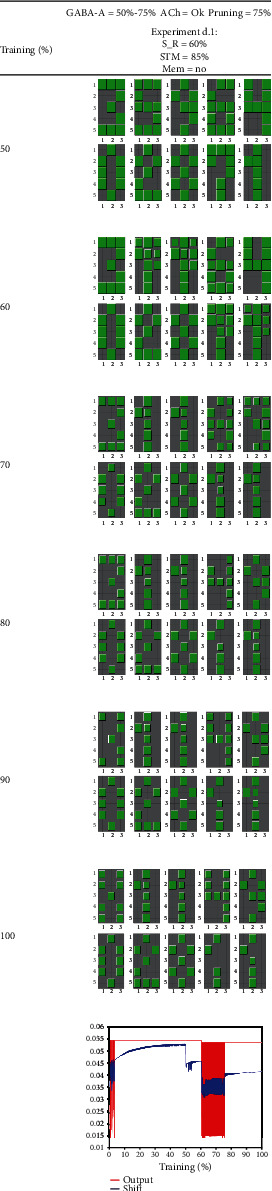
This table presents a case in which GABA-A normalization is recovered despite the usual process of network aging. After the elimination of GABA-A normalization that occurred at 50% of epochs, pattern learning was impaired; that is to say, patterns were poorly recovered, and neurons lost their pattern specificity. With the reduction of sensory stimuli at 60% of epochs, the average shift and output started an intense oscillatory dynamic that suddenly disappeared when GABA-A normalization was reinstalled at 75% of repetitions. After this recovery, the network was capable of learning pattern X, while the recovery of the remaining numerical patterns was damaged. At this later stage, in which many of the connections were pruned, learning is a difficult process requiring much more repetitions than in an intact network. Labels: S_R = sensory reduction; STM = short-term memory; Mem = memantine; ACh = acetylcholine reduction in thalamocortical neurons.

Figures 6–11, will show the different tests performed in the koniocortex model. Each column header will indicate the type of alteration performed in the network and the percentage of epochs when the alteration took place. The meanings of the abbreviations inside the headers are as follows:

- Pruning = 75% means that the elimination of weak connections occurs from 75% of iterations
- GABA − A = 50% means that, at 50% of training epochs, the normalization resulting from GABA-A activity is eliminated
- ACh = 50% means that a reduction of ACh occurs at 50% of repetitions. In computational terms, this means that the sigmoid function stops shifting when parameter *υ* becomes zero
- S_R = 60% means that there is a reduction of sensory stimuli (S_R) at 60% of all repetitions
- STM = 70% means that a short-term memory test (STM) is performed by replacing a randomly selected pattern with the “X” pattern at 70% of the repetitions
- Mem = 55% means that synaptic weight modification is prevented due to the use of memantine (Mem) at 55% of the repetitions

As synaptic pruning is present in every aging person (>75 years of age), all experiments run with pruning. The description of the performed tests and their corresponding parameters are summarized in Table 1.

We repeated each of the experiments 20 times. Since the initial weights and neuron firing thresholds are random, different results are produced until the network stabilizes. This type of variation mainly occurs during the first 100 epochs. After this number of epochs, the experiments evolve similarly and become consistent across repetitions. For this reason, we randomly selected one of the repetitions as a representative of each experiment (see Figures 6–11).

Under each one of the headers, there is a column of blocks. Inside each block, there are ten different numerical patterns. From left to right and from top to bottom, each of the ten numbers represents the patterns that are recalled by the ancillary network when each one of the ten stellate spiny neurons fires. For example, the block that appears in a row labeled 70 represents the ten patterns that are recalled by the ten SS neurons at 70% of repetitions. In this block, for example, the third numerical pattern in the upper row is the pattern recalled by the third SS neuron.

Finally, the bottom curves represent the evolution of the average output and shift of neurons, in terms of the percentage of epochs. These graphs help in the identification of hypermetabolism, which is the preliminary manifestation of AD. Hypermetabolism appears when there are intense and persistent oscillations both in the average output and average shift. Persistent oscillations are associated with a continuous process of allocation and elimination of intrinsic channels in the neuron's membrane.

Now, we proceed to explain the behavior in each column of the tables.

Let us start with Figure 6, which shows the experiments modeling healthy aging. In experiment a.1, we simulate the case of a healthy normal koniocortex in which we blocked neither GABA-A, ACh, nor NMDA. Pruning follows the normal statistical tendency described in the last paragraph of Materials and Methods, thereby starting at 75% of the total epochs. The task of learning a new pattern at 70% of repetitions is successful, as can be seen in the block at 80% in which we substitute a random numerical pattern (in this case number 4) by pattern “X.”

The evolution of the average output (in red) and average shift (in blue) of all modeled neurons appears at the bottom of each column. In the case of healthy aging, the oscillations observed in the output when substituting the pattern are negligible.

In experiment a.2 (also corresponding to healthy aging), stimuli diminish at 60% of the epochs (S_R = 60%). As previously explained, we do this by dividing each component of the numerical patterns by two (pixel value/2). Starting at the block corresponding to 70% of epochs, we see that the patterns recalled by each one of the neurons become fainter. Immediately after stimulus reduction, there is a temporary shift stabilization that quickly leads to a regime of discrete oscillations of the average shift. Although there are a few oscillations during the initial iterations, which is the normal expected behavior, the average output in (red) remains stable in all subsequent iterations.

Let us continue with Figure 7 and Figure 8 showing the experiments that model aging associated with impaired GABA-A receptors. In experiment b.1, we withdrew normalization (due to GABA-A shunting neurons) at 50% of repetitions. In this experiment, there is neither stimulus reduction nor an STM task. This experiment was performed to demonstrate the impact of GABA-A reduction alone, without the presence of other concomitant factors. In this case, the first column in Figure 7 shows an episodic pattern recall impairment from around 60% of epochs. At the same time, the average output (see bottom graph) exhibited a transitory abrupt oscillation that quickly ended due to compensatory factors (intrinsic and synaptic plasticity). Although oscillations ceased, the impairment in patterns' recalling was permanent. This case means that although GABA-A deficit alone does not have catastrophic consequences in terms of hypermetabolism (because oscillations cease), there could be some sequels in terms of memory deficits.

In experiment b.2, besides normalization due to GABA-A withdrawal at 50% of repetitions, stimulus reduction occurred at 60% of epochs. When running the program, we see that at 50% of training, each neuron still processes a different numerical pattern, as expected. From this moment in which GABA-A normalization stopped, patterns' recalling was impaired. Patterns' memorization worsens, and neurons lost their specificity for patterns; that is to say, several neurons fire in front of the same pattern.

Experiment b.3 is shown in the first column of Figure 8 and is similar to the previous one (b.2): each neuron had learned to identify a specific numerical pattern, and, at 50% of epochs, we eliminate the normalization performed by GABA-A. Like in the previous case, the lack of normalization impaired learning so that the univocal correspondence between neurons and patterns was lost. As before, with stimulus reduction at 60% of epochs, abrupt oscillations appeared in the shifts and outputs. Despite this, at 70% of epochs, we applied the task of substituting one of the patterns by pattern X. The network learned pattern X, but the univocal correspondence between patterns and neurons continues impaired. It seems that learning a new pattern contributes to stabilization because the oscillations dampen during pattern X presentation.

Experiment b.4 is like the previous one but with the introduction of an NMDA blocker at 55% of repetitions. Although the network did not forget the numerical patterns, it was not able to learn the new pattern “X” when introduced at 70% of epochs. Differently from the previous case, the presentation of pattern X does not dampen the oscillations.

In summary, an NMDA blocker contributes to preserve older memories but is not capable of preventing the oscillatory dynamics due to stimulus reduction.

The experiments of Figure 9 (c.1 and c.2) and Figure 10 (c.3. and c.4) are designed for testing whether ACh deficit alone is able to produce AD symptoms. Let us recall that the ACh deficit (due to an impairment of the parabrachial nucleus) weakens the preliminary process of input vector separation that takes place in thalamocortical neurons. Experiment c.1 represents a control test for comparison with the next ones. The graph at the bottom of the table shows sparse bursts of oscillations in the outputs and shifts. When we analyzed learning, epoch by epoch, we noticed that during these bursts, the association between neurons and learned patterns is impaired.

In experiment c.2, stimulus reduction is applied at 60% of epochs. In this case, stimulus reduction, instead of increasing the oscillations, seems to dampen them. These sustained oscillations are responsible for the learning impairments seen in the following blocks along training epochs.

In experiments c.3 and c.4, we use the combination of features of experiment c.2 with a short-term memory experiment (STM) for evaluating the use of an NMDA blocker (i.e., memantine). As in previous cases, the STM test consists of substituting a randomly selected pattern by pattern X at 70% of epochs. When this test is done in experiment c.3 without an NMDA blocker application, the NN learned pattern X, but the process was slower and generated intense oscillations. In the experiment c.4, oscillations are abolished when using an NMDA blocker at the expense of not being able to learn pattern X.

Experiments c.1 to c.4 shows that when GABA-A inhibition is normal, ACh deficit produces oscillations that are sparser and weaker than in the case of the association of GABA-A and stimulus reduction. In the cases of ACh deficit, many strategies contribute to abolishing the oscillations, like the NMDA blocker application. Even the reduction of stimuli (that was catastrophic in the case of GABA-A deficit) can help to dampen the oscillations. In contrast (as shown in experiment c.3), STM stimulation can be counterproductive, especially in the absence of memantine. It seems that the cognitive stimulation (STM test) can be either beneficial or harming depending on whether there is either a GABA-A deficit or an ACh deficit, respectively.

The purpose of Figure 11 is to simulate a possible recovery from hypermetabolism when GABA-A signaling returns to normal levels. In this experiment, there was no ACh deficit. Hypermetabolism was manifested by the persistent oscillations of the average shifts and outputs. These oscillations were the consequence of GABA-A deficit followed by stimulus reduction, as shown in examples b.2, b.3, and b.4. At 75% of epochs, GABA-A levels were restored. From this moment, there was a moderate recovery of the patterns recalling capacity, and when an STM test was introduced with pattern X at 85% of epochs, the test was successful. Despite this, the process of synaptic pruning that started at 75% altered the usual course of learning, which, in this case, took longer than in a normal situation and did not end until reaching 100% of epochs. This experiment shows that when GABA-A levels return to normal, there is a real possibility of recovery (although this would depend on the severity of further damage due to beta-amyloid plaque accumulation).

It would be possible to create new alternative computer experiments for testing different therapies like combining NMDA blockers, cognitive stimulation, cholinesterase inhibitors, etc. In these alternative experiments, the correct scheduling of each treatment would be of great importance. Testing these therapies first in the computer and, afterward, with real subjects will be extremely useful and will contribute to the development of an optimal therapeutic strategy.

## 4. Discussion

The experiments presented here show that it is possible to evaluate the evolution of AD with a computational model of a brain structure with learning capabilities under different scenarios. We created these scenarios by combining the effects of neuromodulators, pharmacological drugs, and sensory patterns. In this study, we chose the koniocortex because, as mentioned in Introduction, AD begins in sensory cortices [27–29] (although appearing in its mildest form). Besides, this structure is directly associated with sensory stimuli, which we were interested in assessing. Another advantage of the modeled koniocortex is that it exhibits successful emergent learning so that we can test the effects of AD along with a learning task.

As seen in Results, we decided to exhibit neurons' average output and their firing thresholds (shifts) over 2,000 training epochs (as mentioned, the presentation of the whole set of numbers constitutes one epoch).

We also presented the appearance of the memorized patterns over epochs. In this way, we displayed three crucial variables related to AD: first, the red curves allow us to study neurons' average firing probability and identify hypermetabolism. Secondly, the blue curves enable us to see the shifting of the firing threshold and infer the amount of intrinsic channel allocations in neurons' membranes (the smaller the shift, the higher the number of intrinsic channels). Thirdly, the appearance of memorized patterns over the epochs allows us to monitor long and short-term memory in the network. We assessed these variables by altering the input stimuli or simulating a treatment. Our interest in monitoring the possible oscillatory activity in the average firing and shift related to hypermetabolism is because hypermetabolism is the prelude to the more harmful consequences of AD: *β*-amyloid plaque creation and memory impairments. Hypermetabolism would be the consequence of continually placing and deleting intrinsic channels when the neuron's firing threshold (activation function's shift) tries unsuccessfully to reach an equilibrium point. Although hypermetabolism and neuronal activity are related concepts, hypermetabolism can be easily monitored in human patients using PET and MRI scans.

The main result derived from our model is that, although stimulus reduction is innocuous in a young, healthy brain, it ignites hypermetabolism in a brain with GABA-A signaling deficits. ACh impairments are also able to produce hypermetabolism more moderately. This is because we discovered that GABA-A and ACh are involved in computational processes that facilitate pattern normalization and pattern separation, respectively, during the learning processes performed in the brain [26]. In this way, disruption of the algorithms involved in learning processes in the brain seems to be at the origin of the destructive processes of AD. Regarding learning, brain structures like the koniocortex adjust their synaptic weights so that they acquire stable values through homeostatic processes in which IP compensates for weight variations [22]. In a previous article, we suggested that impairments in IP are a crucial factor for the onset of AD [91]. The importance of IP in learning and AD has been corroborated by Dunn and Kaczorowski [92]. Calcium homeostasis processes underlying AD are also discussed in Popugaeva et al. [93].

The explanation behind the oscillatory behavior triggered when stimulus reduction takes place in a GABA-A-deficient brain is that GABA-A shunting basket neurons produce a sort of pattern normalization (see Section 1.3 and explanation of Equation (A.3) in the appendix) over incoming sensory patterns. When these basket neurons correctly perform their normalizing function, the resulting computation is as if all sensory patterns were always similar in size. In this case, GABA-A represents a protective factor that acts even in the case of reduced stimuli (which, due to GABA-A, are resized, i.e., normalized). When the protective factor of GABA-A is not present, reduction of stimuli (like in macular degeneration, deafness, and retirement) triggers a process in which neurons lower their firing threshold through IP to adequately respond to weaker stimuli (recall explanations of Equations (A.6) and (A.7) in the appendix). For this process, extra intrinsic channels are allocated in the cellular membrane, having this process a high metabolic cost. Although in an ideal situation, neurons would adjust their firing thresholds until they gradually reach a new lower threshold, in our simulations, these adjustments take place in an oscillatory manner. In this case, firing threshold adjustments take place along a continuous recurrent process of allocation and elimination of intrinsic channels. Repeating these processes, thousands of times would produce a much higher cost in metabolic terms than when intrinsic channels are simply gradually allocated.

During intrinsic channel allocation processes, the intense firing of neurons is another process that contributes to enhancing hypermetabolism. Besides impaired neural homeostatic processes, intense firing has been considered another cause of *β*-amyloid plaque deposition [94, 95]. Thus, the preemptive usage of low doses of antiepileptic drugs like diazepam [96] and levetiracetam [97] has been shown to have a neuroprotective effect in AD.

When instead of a GABA-A deficit, there is ACh depletion, the preliminary process of input vector separation is affected, as explained in Figure 4. Without this vector's separation process, the subsequent WTA operation requires more epochs to be accomplished. This is manifested in the short-term memory test performed in experiment c.3, in which the modest oscillations associated with ACh deficits grew significantly. It seems that in this case, cognitive stimulation is counterproductive and could contribute to exacerbating hypermetabolism. In experiment c.4, we see that by using an NMDA blocker, oscillations completely disappear at the expense of not being capable of learning the new pattern X. This fact could justify the success of treatments combining NMDA blockers (memantine) and acetylcholinesterase inhibitors (donepezil) [98] in a background of milder oscillations related to an ACh deficit [99].

In the case of GABA-A deficit, the oscillations that appear after stimulus reduction are much higher in frequency (see curves in experiments b.2, b.3, b.4, and d.1) than those related to ACh impairment (experiments c.2 and c.3). The existence of a relationship between GABA interneuron malfunction and intense neural oscillatory activity was pointed out by Verret et al. [100] when working in AD animal models. Regarding a possible AD treatment, experiment d.1 is designed to test whether the complete recovery of GABA-A functionality at 75% of epochs can stop the oscillations. We see that not only the intense oscillatory activity completely disappears but also the learning capability of the network is wholly recovered so that it was possible for the network to learn a new pattern (pattern X) that was presented at 85% of computer iterations (in Figure 11, the tiles in green show that this pattern was learned entirely at 100% of epochs by number one stellate neuron).

These computational results support recent pharmacological studies focusing on GABA-A neurons' protecting drugs [101–103]. Experiment a.2 shows that when GABA-A functionality is preserved, no oscillations take place even in the case of stimulus reduction. As mentioned in Section 1.3.3, the ingestion of nutrients like fisetin (strawberries, tomatoes, oranges, and cucumber) and terpenoids (salvia, peppermint, ginger, Curcuma longa, cinnamon, cloves, and mustard) collaborates in the synthesis and expression of GABA-A receptors.

Although the results of the present experiment are encouraging, we should acknowledge, however, that we assessed AD by modeling a particular brain structure and that other brain structures related to learning also deserve evaluation. It is possible that the same computational process that seems impaired in the koniocortex, i.e., pattern separation and pattern normalization, might also be defective in other parts of the brain, thereby generating an abnormal oscillatory dynamic. These prospective assessments performed in more comprehensive models might contribute to support our hypothesis that AD is a consequence of stimulus reduction in a brain with GABA-A deficit. Although, as mentioned in Introduction, we prefer to perform such tests on models whose functionality is clear to us, we do not underestimate the potential of existing realistic models of the entire brain [14, 17]. We believe that the most significant drawback presented by these comprehensive models is that most of them surprisingly fails to use the property of IP. We believe that by incorporating IP into these models, new functionalities would emerge from them, such as learning and pattern completion capabilities, in such a way that they could also be used to model the cognitive impairments of AD.

## 5. Conclusions

As mentioned in Introduction, AD is a multifaceted illness in which the factors involved are usually separately analyzed. In this work, we combine many of these factors, interacting dynamically inside a computational model of the cerebral koniocortex, the first cortical relay station to process sensory information, and also one of the early nervous system structures that are affected by AD.

We tested this hypothesis with the koniocortex model engaged in the task of learning ten numerical patterns. The model underwent a short-term memory test by substituting one random pattern by pattern X in the middle of learning. Another situation performed with the koniocortex simulation was to stop the synaptic weight adjustment as when using an NMDA blocker. During these scenarios, we assessed whether the network exhibits hypermetabolism, which is a common feature during the initial stages of AD. Due to the possibility of an increment in computational complexity, instead of metabolism, we evaluated a related measurement: the average output of all koniocortex neurons.

The computation shows that the onset of AD is related to a reduction of sensory/cognitive stimuli (like in deafness, macular degeneration, or retirement) when there is a preexistent deficit in GABA-A. In computational terms, the lack of pattern normalization due to the divisive inhibition of basket neurons leads to an oscillatory behavior of the neurons' outputs and their firing thresholds. In real neurons, the continuous adjustment of the neuronal firing threshold by allocating and eliminating intrinsic ion channels exhausts neurons' metabolic resources, driving them to the phase of beta-amyloid plaque deposition that is beyond the scope of our study.

Although our hypothesis should be tested in animal models and, afterward, with real patients, we suggest that in the meantime, for preserving elders' health, their caregivers should be on alert in front of scenarios that contribute to the reduction of the sources of stimuli. A confinement situation for epidemic contention could be a nowadays example of these scenarios. In this type of situation, it would be desirable to look for alternative sources of cognitive and sensory stimuli for this group of people.

## Figures and Tables

**Figure 12:**
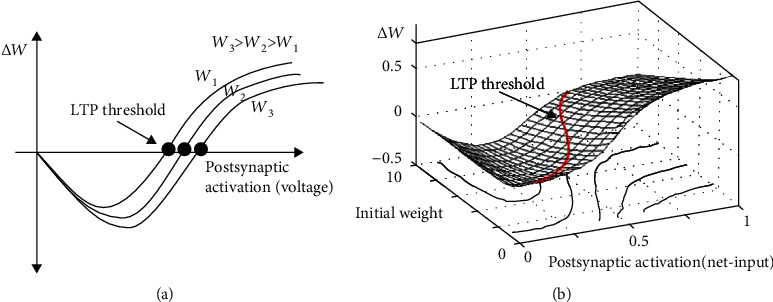
(a) Shape of curves obtained with real neurons for different initial synaptic weights *w*_*i*_. In this case, the experiment consisted of injecting current in the presynaptic neuron and measuring the postsynaptic voltage. The point where curves cross the horizontal axis is called the long-term potentiation threshold. (b) Family of curves obtained in the computer model through the presynaptic rule (adapted from Figure 2 in [105]). Both graphs exhibit metaplasticity: the rightward elongation of the curves along the horizontal axis for higher values of initial synaptic weights. For a more detailed explanation of how the curves were obtained, we suggest the reader to study References ([105] (Section 2), ([22] (Section3.2)).

**Figure 13:**
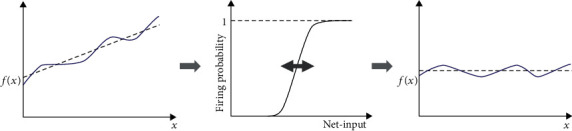
When a time series *f*(*x*) is placed at the single input of a neuron with intrinsic plasticity property, the output of the neuron is the same time series *f*(*x*) but with its moving average removed.

**Table 1:** Description of the performed tests and their corresponding parameters. Abbreviations: (a) GABA-A: this column shows the percentage of epochs in which GABA-A deficit initiates. (b) ACh: this column exhibits the percentage of epochs in which ACh deficit begins. (c) Pruning: the values indicate the percentage of epochs in which pruning initiates. (d) S_R: the values below this title indicate the percentage of epochs in which stimulus reduction starts. (e) STM: the values below this title indicate the percentage of epochs in which the experiment of short-term memory initiates. (f) Mem: when memantine treatment (i.e., an NMDA blocker simulation) is applied, the numeric value shows the percentage of epochs in which memantine treatment initiates.

		Parameters					
Test	Experiment	GABA-A	ACh	Pruning	S_R	STM	Mem
Normal aging	a.1 Test of short-term memory	Ok	Ok	75%	No	70%	No
	a.2 Reduction of sensory stimuli and short-term memory test	Ok	Ok	75%	60%	70%	No

Aging with impaired GABA-A receptors	b.1 No sensory reduction	50%	Ok	75%	No	No	No
	b.2 Reduction of sensory stimuli	50%	Ok	75%	60%	No	No
	b.3 Reduction of sensory stimuli and short-term memory test	50%	Ok	75%	60%	70%	No
	b.4 Reduction of sensory stimuli, short-term memory test and administration of an NMDA receptor blocker (memantine)	50%	Ok	75%	60%	70%	55%

Aging with ACh deficiency	c.1 No sensory reduction	Ok	50%	75%	No	No	No
	c.2 Reduction of sensory stimuli	Ok	50%	75%	60%	No	No
	c.3 Reduction of sensory stimuli and short-term memory test	Ok	50%	75%	60%	70%	No
	c.4 Reduction of sensory stimuli, short-term memory test and administration of memantine	Ok	50%	75%	60%	70%	55%

Aging with GABA-A impairment at 50% of epochs. Recovery at 75% of epochs	d.1 Sensory stimulus reduction, short-term memory stimulus, and recovery of GABA-A receptor functionality. Pruning is present as in previous experiments modeling healthy brain aging	50%-75%	Ok	75%	60%	85%	No

**Table 2:** Here, we list the computational processes performed by each type of neuron in each layer of the koniocortex model. The consequences of several lesions (due to age) in these different layers are also mentioned. When these lesions coincide with a reduction of sensory stimulation, we expect a further degree of deterioration that resembles AD. In the last column, we enumerate some pharmacological treatments to counteract the failures listed in previous columns.

Type of neurons in each layer	Computational process	Age-dependent damage	Computational failure due to aging	Expected failure due to sensory loss & aging	Pharmacological treatment
Basket neurons	Neural competition	Neural loss, GABA reduction	Discrimination deficits	Discrimination deficits	

Spiny stellate neurons	Patterns learning in synaptic weights	Pruning of synaptic connections	Problems in novel pattern learning	Hypermetabolism, forgetting, learning problems	NMDA blockers avoid forgetting but preclude new learning

Shunting basket neurons	Normalization	GABA-A synthesis reduction	Lack of normalization	Hypermetabolism, difficulty in novel pattern learning	

Thalamocortical neurons	Input patterns' separation	ACh deficit affects intrinsic plasticity	Failure in pattern separation processes	Slow or inefficient learning	DSE inhibitors increase acetylcholine

Input sensory neurons	Input detection	Damage of sensory receptors	Sensory deficit + GABA deficit ⟶ AD	Sensory stimuli contrast deficits	

Parabrachial acetylcholine neurons	Boost of intrinsic plasticity and patterns' separation in TC neurons	Cholinergic neuron degeneration	Problems in patterns' separation	Problems in patterns' separation	Acetylcholinesterase inhibitors increase acetylcholine

## Data Availability

The computational codes used in this manuscript are available at https://osf.io/hkx9w. This material included the MATLAB code used to support the findings of this study.

## Appendix

### Mathematical Foundations of Koniocortex Network

In this section, we describe the equations used in the koniocortex model in which the neurons' output yields a probability value ranging from 0 to 1. For simplicity, we do not model neurons' axon cable phenomena.

The koniocortex is a competitive model in which the neuron's net-input is calculated by a vector projection. For this calculation, we compute the inner product of weight vectors and the normalized input patterns (lowercase notation meaning vector normalization):

(A.1) $$\begin{array}{c} \vec{i}=\frac{\vec{I}}{\left\|\vec{I}\right\|}. \end{array}$$

The type of normalization used here is the l1-norm:

(A.2) $$\begin{array}{c} \left\|\vec{I}\right\|=\overset{n}{\underset{i=1}{\sum }}\left|I_{i}\right|. \end{array}$$

One way of interpreting neurons' weights is as if they were the components of a vector prototype ${\vec{T}}^{j}$ so that ${\vec{T}}^{j}={\vec{W}}^{j}=\left[W_{j1},W_{j2},\cdots ,W_{jn}\right]$. In this way, the net-input of neurons can be calculated as $\text{ne}{\text{t}}_{j}=\left\|{\vec{W}}^{j}\cdot \vec{i}\right\|=\left\|{\vec{T}}^{j}\cdot \vec{i}\right\|=\left\|{\vec{T}}_{\vec{I}}^{j}\right\|$ which is the projection of prototype $\;{\vec{T}}^{j}$ over the ongoing input pattern. Although in NNs models the net-input value is dimensionless, it is conceptually equivalent to the postsynaptic activation that in electrophysiology is measured in volts.

In the case of spiny neurons (which undergo competition), we calculate this same result another way:

(A.3) $$\begin{array}{c} \text{ne}{\text{t}}_{j}=\frac{\left\|{\vec{W}}^{j}\cdot \vec{I}\right\|}{\left\|\vec{I}\right\|}, \end{array}$$

where the numerator is the weighted input from excitatory thalamocortical neurons. The denominator corresponds to the operation produced by a shunting basket neuron when performing a sequence of two operations (a) the calculation of an l1-norm, $\left\|\vec{I}\right\|$, by summing the thalamocortical inputs and (b) placing this result in the denominator according to the shunting/divisive type of inhibition of GABA-A neurons.

The incremental version of the presynaptic rule was used in the koniocortex model for altering synaptic weights:

(A.4) $$\begin{array}{c} \Delta W=\xi I\left(O-W\right), \end{array}$$

where *O* and *I* are postsynaptic and presynaptic action potential probabilities and *ξ* is a small positive constant, the so-called “learning factor.” This learning factor value was set to 0.0019. Synaptic plasticity freezing due to an NMDA blocker was simulated by setting this value to 0.

The plasticity curves yielding the variation of synaptic weight in terms of postsynaptic voltage that were empirically obtained by Artola et al. [104] (see Figure 12(a)) are very similar to the curves depicted in Figure 12(b) obtained by using the presynaptic rule equation [22, 105]. The presynaptic rule also models metaplasticity [106, 107], a property of biological synapses that elongates the plasticity curves rightwards for higher initial synaptic weights, as shown in both graphs of Figure 12.

The function that relates the neuron's output in terms of its postsynaptic activation can be modeled in different ways (like a linear, sigmoidal, and Gaussian function). In the case of the koniocortex, inhibitory neuron activation functions were modeled as linear functions. For modeling sensory, thalamocortical, and spiny neurons, we used the following equation yielding the output of the neuron, *O*_*j*_, in terms of its net-input (postsynaptic activation):

(A.5) $$\begin{array}{c} O_{j}=\frac{1}{1+e^{-k\left(\text{ne}{\text{t}}_{j}-s_{j}\right)}}, \end{array}$$

where *s*_*j*_ is the shift of the activation function ranging from 0 to 1 and *k* is a steepness factor whose value depends on the type of neurons.

Biological neurons exhibit a property called intrinsic plasticity (IP) [32]. According to this property, the sigmoidal activation function gradually shifts rightwards or leftwards, leveling the activation of highly or scarcely activated neurons, respectively (see Figure 1). The so-called shift parameter *s*_*j*_, ranging from zero to one, is used to model the sigmoidal curve rightward shift. The shift value corresponds to the steepest point of the sigmoid curve and is equivalent to the classical concept of “firing threshold.” We can formulate the activation function as

(A.6) $$\begin{array}{c} O_{j}=f\left(\left\|{\vec{T}}_{\vec{I}}^{j}\right\|,s_{j}\right). \end{array}$$

According to Desai ([33], p.398), the activation function

relates a neuron's output (its firing rate) to its input (the synaptic current it receives). If the input is too low, the cell will hardly ever fire, because of the spike threshold [The firing threshold.]; if it is too high, the firing rate will saturate, because there is some physical limit on how fast a neuron can fire. Between the two is a sensitive region, where the neuron's output is a function of its inputs. One strategy for dealing with fluctuations in input – caused, for example, by synapse formation or Hebbian potentiation – is to shift the position or slope of the f–I curve so that the sensitive region always corresponds well with average input.

We proposed the following equation [22] for calculating the shift of the activation function, *s*, at time *t* in terms of the shift and output probability of the neuron at time *t*‐1:

(A.7) $$\begin{array}{c} s_{t}=\frac{\upsilon \cdot O_{t-1}+s_{t-1}}{\upsilon +1}, \end{array}$$

where *υ* is a small factor that adjusts the shifting speed of the activation function. Its value is 0.0199 in the koniocortex simulation. The initial shift, *s*, was 0.061 in all neurons. When neurons are highly activated, the tendency of the shift factor, *s*, is to increase, thereby shifting the activation function rightwards. When neurons are less activated, the tendency of the shift factor, *s*, is to decrease, so that the activation function is shifted leftwards (see Figure 1).

One compelling case that occurs in thalamocortical neurons refers to a single input neuron, where IP contributes to removing the moving average of a series of input values ([26], section 2), making the neuron behave like a high-pass filter (see Figure 13).

When, instead of a single neuron, there is a matrix of neurons receiving patterns (like the case of thalamocortical neurons), subsequent patterns undergo the subtraction of their moving average (which is also a pattern), thereby making patterns more separated from each other, like in Figure 4. This input vector separation effect stops when we eliminate the movement of the sigmoid by zeroing factor *υ*. In the real koniocortex, this happens when ACh neurons are blocked or die, thereby losing their boosting effect over intrinsic thalamocortical plasticity.
